# A COVID‐19 CXR image recognition method based on MSA‐DDCovidNet

**DOI:** 10.1049/ipr2.12474

**Published:** 2022-03-15

**Authors:** Wei Wang, Wendi Huang, Xin Wang, Peng Zhang, Nian Zhang

**Affiliations:** ^1^ School of Computer and Communication Engineering Changsha University of Science and Technology Changsha China; ^2^ School of Electronics and Communications Engineering Sun Yat‐sen University Shenzhen China; ^3^ Department of Electrical and Computer Engineering University of the District of Columbia Washington DC USA

## Abstract

Currently, coronavirus disease 2019 (COVID‐19) has not been contained. It is a safe and effective way to detect infected persons in chest X‐ray (CXR) images based on deep learning methods. To solve the above problem, the dual‐path multi‐scale fusion (DMFF) module and dense dilated depth‐wise separable (D3S) module are used to extract shallow and deep features, respectively. Based on these two modules and multi‐scale spatial attention (MSA) mechanism, a lightweight convolutional neural network model, MSA‐DDCovidNet, is designed. Experimental results show that the accuracy of the MSA‐DDCovidNet model on COVID‐19 CXR images is as high as 97.962%, In addition, the proposed MSA‐DDCovidNet has less computation complexity and fewer parameter numbers. Compared with other methods, MSA‐DDCovidNet can help diagnose COVID‐19 more quickly and accurately.

## 1 INTRODUCTION

The 2019‐nCoV is spreading with an extremely fast rate. Coronavirus disease 2019 (COVID‐19) caused by 2019‐nCoV has put many countries and regions with scarce medical resources and low medical standards into trouble. The most common used method for diagnosing COVID‐19 is a detection method based on reverse transcriptase polymerase chain reaction (RT‐PCR). It has high specificity, but the current demand for detection kits is increasing [1]. In addition, its sensitivity is low, which makes it prone to false negative diagnostic results. False negative results have serious consequences on the COVID‐19 prevention. For countries and regions where medical resources are scarce, a fast, reliable, and low‐cost detection method should be sought. CXR is the most widely used imaging test to diagnose heart and other chest diseases [2]. Compared with CT scans, CXR is more popular, and X‐rays have lower ionizing radiation [3].

Detecting diseases through chest radiographs is an extremely challenging task. It requires a certain amount of professional knowledge and careful observation. COVID‐19 contains some radiological features that can be detected by CXR. However, if these characteristics are analysed by manual film reading, not only will it take up a lot of medical staff's time, but it will also be prone to errors due to visual fatigue and other disturbances. Therefore, it is necessary for us to find a way to automate the detection of CXR.

The purpose of this study is to search a lightweight and accurate CXR image automatic recognition method of COVID‐19 to assist medical staff in diagnosis. Since convolutional neural networks (CNNs) have excellent performance in image recognition task, especially in image classification task, CNN model is considered to realize this method. In order to ensure that the model can accurately identify the CXR image of COVID‐19 in a low‐cost way, deep separable convolution [4], feature reuse and multi‐scale feature fusion are adopted fully when designing the network structure.

The remainder of the paper is arranged as follows: Section 2 discusses the related work of CNN image recognition and medical image recognition. Section 3 describes the structure of our proposed network and its modules. Section 4 shows the experimental dataset, parameter setting and experimental results and analyses the results in detail. Section 5 carefully analyses the advantages of the structure of MSA‐DDCovidNet and the limitations of the study. Section 6 summarizes the paper and describes our prospects for the future of this study.

## 2 RELATED WORK

In recent years, deep learning has been widely used in medical image detection. For example, Wang W et al. [5] applied the image classification method based on Deep Learning to the classification of Colonic Polyps and proposed the improved approaches VGGNets‐GAP and ResNets‐GAP with global average pooling (GAP) to classified colonoscopy polyp images for assisted diagnosis. Inspired by the DenseNet [6] and MobileNet [4], Wang W et al. [7] proposed Dense‐MobileNet, which got a good performance in children's colonoscopy polyp dataset. As a representative branch of deep learning technology, convolutional neural network (CNN) has excellent performance in image feature extraction and learning [8]. Therefore, researchers recommend using deep learning technology to help detect lesion information on CXR images, save medical resources, and improve diagnosis efficiency. For example, Khan et al. [9] proposed the CoroNet based on the structure of Xception [10], which achieved good performance on the COVID‐19 CXR image classification. Based on Xception [10] and ResNet50V2 [11], Rahimzadeh et al. [12] designed a network which improved the performance of the network by combining the output feature of the two networks. The network has achieved good results on a dataset containing three types of CXR images of COVID‐19, pneumonia and normal. Wang et al. [13] designed the channel feature weight extraction module (CFWE) according to the characteristics of CXR image and proposed a new CFW‐Net. Ozturk et al. [14] proposed a DarkCovidNet, which was improved based on the DarkNet‐19 network and achieved good classification accuracy. To recognize the COVID‐19 CXR images, Wang et al. [15] designed a new network MCFF‐Net based on the Parallel Channel Attention Feature Fusion Module (PCAF). Wang et al. [16] proposed a new method to detect COVID‐19 patients in CXR images based on MAI‐Nets, and finally got an excellent result with an accuracy of 96.42%.

## 3 ARCHITECTURE DESIGN

Commonly, CXR images of different classes are highly convergent, and CXR images in the same class have low specificity. This leads to model deviation and overfitting, which reduces the performance and generalization of the model. Moreover, CNN for mobile terminals requires a model with few parameters and fast speed, otherwise it will cause delays and undermine recognition efficiency. In response to the above problems, a new lightweight CNN, MSA‐DDCovidNet, is proposed, based on DMFF module and D3S module and the multi‐scale spatial attention (MSA) mechanism.

### 3.1 DMFF module and D3S module

**FIGURE 1:**
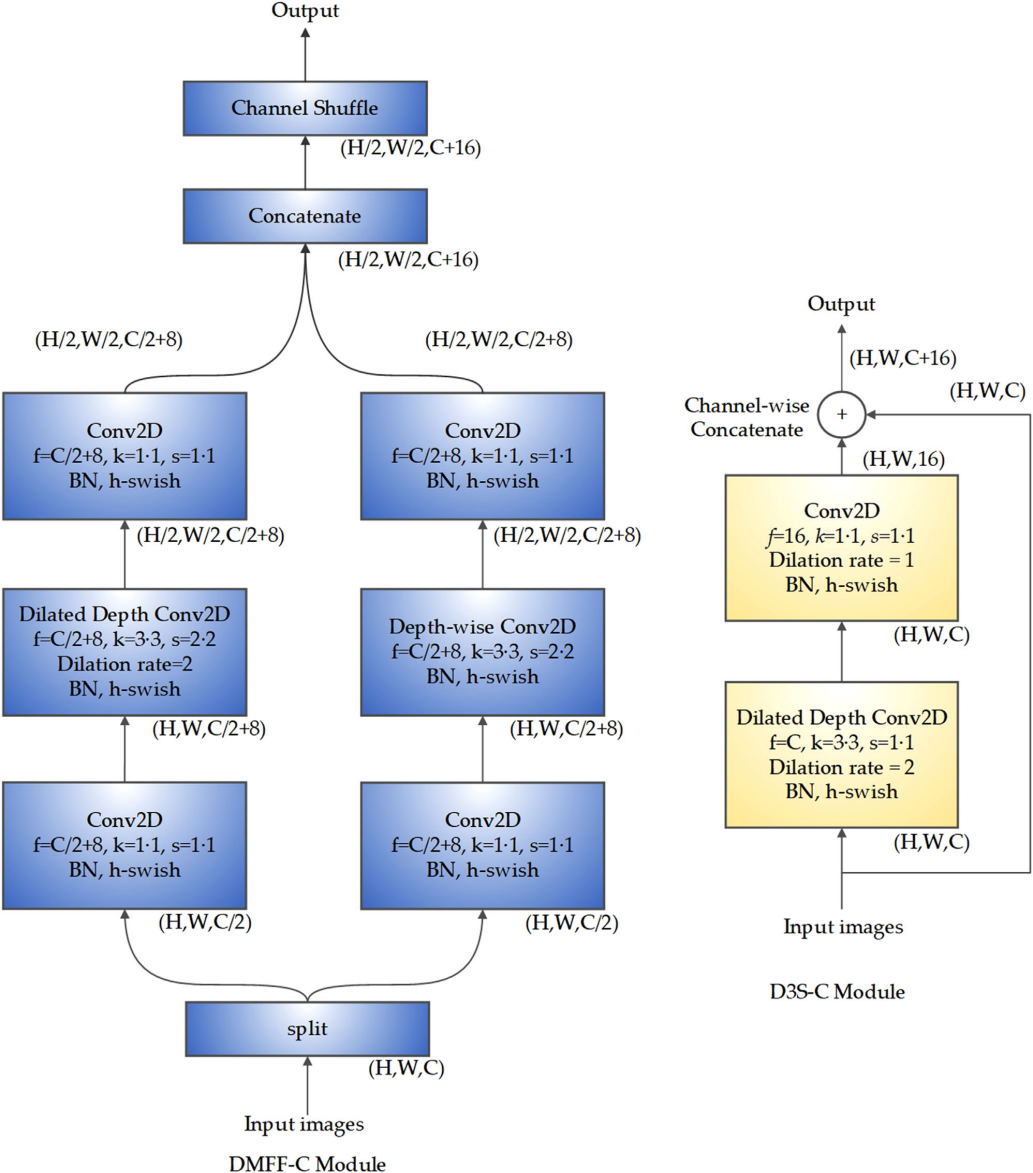
The structure of DMFF Module and D3S Module

**FIGURE 2:**
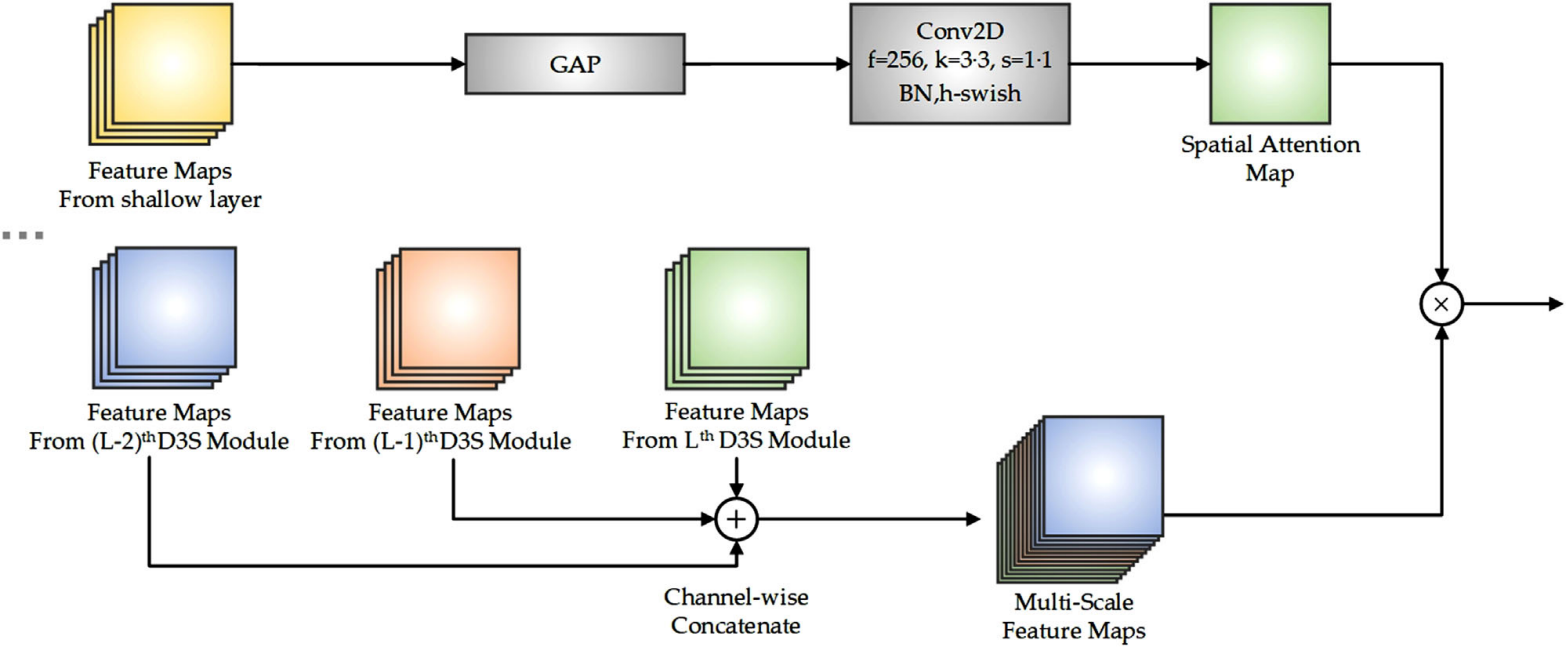
The structure of Multi‐scale Spatial Attention mechanism

The DMFF module and the D3S module are innovatively proposed by our team, and both are modules based on deep separable convolution. They have high computational efficiency and have strong representational capacity on the shallow and deep feature maps respectively. Their structure diagrams are shown in Figures 1 and 2. In Figures 1 and 2, *H*, *W*, and *C* denote the height, width, and channels of the feature maps, respectively; *f* means the number of convolution kernels, *k* represents the size of the convolution kernel, and *s* denotes the step size. Depth Separate convolution decomposes the convolution process into two processes: depth‐wise convolution and point‐wise convolution. Such decomposition process can greatly reduce the amount of calculation and model parameters. Applying h‐swish can alleviate the delay [17], so h‐swish is adopted as the activation function in the network.

DMFF module splits the input feature maps into channels and generates two branches. After increasing the channels with point‐wise convolution, one of branches uses the dilated depth‐wise convolution layer, that is, the depth‐wise convolution layer using dilated convolution kernel with an expansion rate of 2, instead of using the ordinary convolution kernel. The other branch uses depth‐wise convolutional layer after a point‐wise convolution layer. Finally, it concatenates the channels of feature maps of two branches, and gets the output after a channel shuffle [18] operation. Obviously, the receptive fields of the two branches are different. The channel‐wise concatenate operation can realize multi‐scale feature fusion and enhance the spatial representational capacity of the model. Since the dilated convolution with an expansion rate of 2 does not increase the complexity of the model [11], the parameters and the amount of calculation of the two branches are the same. Since the features extracted by the convolutional layer close to the input contain detailed texture information, the DMFF module will be used in the shallow layers of the proposed network.

D3S module is based on dilated depth‐wise separable convolutional layer and dense connection. The input feature maps pass through a dilated depth separable convolutional layer, and then the obtained feature maps and the input feature maps are channel‐wise concatenated as the output of the module. Compared with standard convolution, the dilated depth‐wise separable convolution has fewer parameters and calculation, and a larger receptive field, which makes the model more lightweight and efficient. The features extracted from the deep layers of the network are more critical for distinguishing heterogeneous samples. Feature reuse can alleviate information loss. Therefore, the D3S module will be used in the deep layers of the proposed network.

### 3.2 Multi‐scale spatial attention (MSA) mechanism

Inspired by Kim et al. [19], a novel multi‐scale spatial attention (MSA) mechanism is proposed. Before being input to the fully connected layer, the feature map will be input to MSA attention, as shown in Figure 2. Let there be *L* successive D3S modules in the network. On the one hand, to obtain a spatial attention map, the feature maps output by the first DMFF module will be input to a global average pooling layer and a standard convolution layer. The resulting feature maps are token as a spatial attention map; on the other hand, three groups of feature maps containing different depth semantic features are channel‐wise concatenated. Such resulting feature maps contains rich multi‐scale deep features. These feature maps are multiplied with the spatial attention map to extract the key spatial information in the feature map. Compared with the single‐scale spatial attention mechanism, MSA mechanism can capture feature information of different depths, and has better spatial representational capacity.

### 3.3 The structure of MSA‐DDCovidNet

**FIGURE 3:**
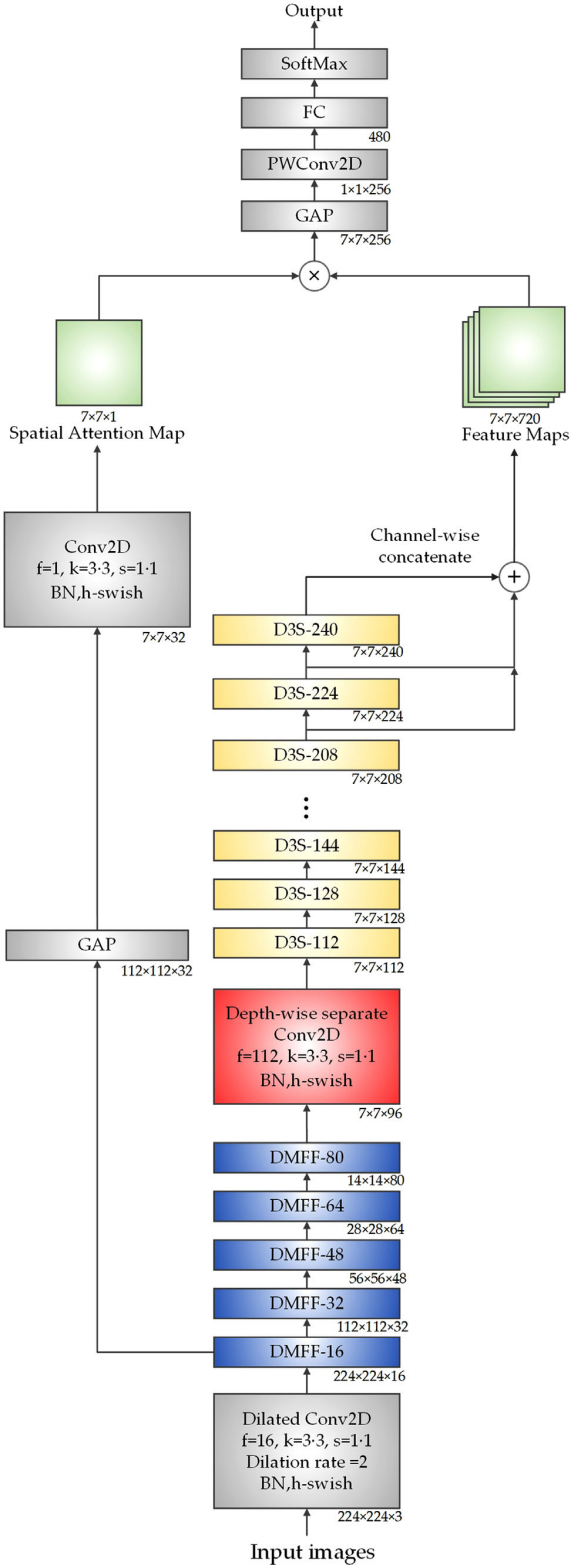
The structure of MSA‐DDCovidNet

The structure of MSA‐DDCovidNet is shown in Figure 3. The input image is preprocessed before being input to the model. The first layer contains a dilated convolution filters with an expansion rate of 2. Then the DMFF module is used for five times to halve the spatial dimension (the height and width) of the feature maps, remove redundant information and compress the features. And then the depth‐wise separable convolution layer is designed to enrich feature information. Next, nine successive D3S modules are set to extract deep features and alleviate the disappearance of gradients. Then the MSA mechanism is used to extract the spatial domain information in the multi‐scale feature maps. After the global average pooling layer, the spatial size of the feature maps becomes 1 × 1. Then a point convolution layer is used to increase the feature dimension and full connection layer. Next, a fully connected layer is used to reduce the impact of feature coordinate information on classification. Finally, the SoftMax layer is used for classification.

### 3.4 Network complexity

In this work, the amount of computation and the number of parameters are adopted to measure the complexity of the model. The parameters generated by the weight layers in CNN, which mainly includes convolution layer and full connection layer. The amount of computation refers to floating‐point operations (FLOPs). All kinds of operations in the network will produce computation, even a simple element‐wise addition operation. The parameters and the amount of computation of the model are mainly related to the depth, width, the resolution of input images and the structure of model.

For a given input feature map H_i_ · W_i_ · C_i_ and the output feature map H_o_ · W_o_ · C_o_, the parameters P_std_ and the amount of computation F_std_ produced by a standard convolution are as follows:

(1) $$P_{\mathrm{std}}=C_{i}\cdot k^{2}\cdot C_{o}$$

(2) $$F_{\mathrm{std}}=H_{o}\cdot W_{o}\cdot k^{2}\cdot C_{i}\cdot C_{o}$$

Since the dilated convolution with an expansion rate of 2 will not increase parameters and calculations, the parameters P_dw_ and the amount of computation F_dw_ generated by a depth‐wise convolution and a depth‐wise dilated convolution process with an expansion rate of 2 are as follows:

(3) $$P_{\mathrm{dw}}=C_{i}\cdot k^{2}$$

(4) $$F_{\mathrm{dw}}=H_{o}\cdot W_{o}\cdot C_{i}\cdot k^{2}$$

For a given input feature map H · W · C and the output feature map (H / 2) · (W / 2) · (C + 16), the parameters P_conv3_1_ and the amount of computation F_conv3_1_ generated by a standard convolution with kernel size 3 × 3 are as follows:

(5) $$P_{\mathrm{conv}3\_1}=9\cdot C\cdot \left(C+16\right)$$

(6) $$F_{\mathrm{conv}3\_1}=9\cdot \left(H/2\right)\cdot \left(W/2\right)\cdot C\cdot \left(C+16\right)$$

And when the DMFF module is used to complete the above dimension conversion, the parameters P_DMFF_ and the amount of computation F_DMFF_ generated by a DMFF module are as follows:

(7) $$P_{\mathrm{DMFF}}=C^{2}+33\cdot C+272$$

(8) $$F_{\mathrm{DMFF}}=H\cdot W\cdot \left(\frac{5}{8}\cdot C^{2}+\frac{57}{4}\cdot C+68\right)$$

Therefore, compared to a standard convolution, the reduction in parameter ∆_DMFF_P_ and computation ∆_DMFF_F_ achieved by DMFF module is shown as follows:

(9) $$\Delta_{\mathrm{DMFF}\_P}=P_{\mathrm{conv}3\_1}-P_{\mathrm{DMFF}}=8\cdot C^{2}+111\cdot C-272$$

(10) $$\begin{array}{c} \Delta_{\mathrm{DMFF}\_F}=F_{\mathrm{conv}3\_1}-F_{\mathrm{DMFF}}=H\cdot W\cdot \left(\frac{13}{8}C^{2}+\cdot C-68\right) \end{array}$$

Similarly, for a given input feature map H · W · C and the output feature map H · W · (C + 16), the parameters P_conv3_2_ and the amount of computation F_conv3_2_ generated by a standard convolution with kernel size 3 × 3 are as follows:

(11) $$P_{\mathrm{conv}3\_2}=9\cdot C\cdot \left(C+16\right)$$

(12) $$F_{\mathrm{conv}3\_2}=9\cdot H\cdot W\cdot C\cdot \left(C+16\right)$$

When the D3S module is used to complete the above dimension conversion, the parameters P_D3S_ and the amount of computation F_D3S_ generated by a D3S module are as follows:

(13) $$P_{D3S}=9\cdot C+16\cdot C=25\cdot C$$

(14) $$F_{D3S}=9\cdot H\cdot W\cdot C+16\cdot H\cdot W\cdot C=25\cdot H\cdot W\cdot C$$

Therefore, compared to a standard convolution, the reduction in parameter ∆_D3S_P_ and computation ∆_D3S_F_ achieved by D3S module are shown as follows:

(15) $$\Delta_{D3S\_P}=P_{\mathrm{conv}3\_2}-P_{D3S}=9\cdot C^{2}+119\cdot C$$

(16) $$\begin{array}{c} \Delta_{D3S\_F}=F_{\mathrm{conv}3\_2}-F_{D3S}=9\cdot H\cdot W\cdot C^{2}+119\cdot H\cdot W\cdot C \end{array}$$

Obviously, ∆_DMFF_P_ > 0, ∆_DMFF_F_ > 0, ∆_D3S_P_ > 0 and ∆_D3S_F_ > 0, which means DMFF module and D3S module make positive contribution to reduce the parameters and calculation.

The complexity of MSA mechanism is analysed. For three sets of input feature map with shapes H · W · C, H · W · (C + 16), H · W · (C + 32) input feature map, the output feature map H · W · (3 · C + 48) and the shallow feature map H_1_ · W_1_ · C_1_, the parameters P_MSA_ and the amount of computation F_MSA_ generated by MSA mechanism are as follows:

(17) $$P_{\mathrm{MSA}}=9\cdot C_{1}$$

(18) $$F_{\mathrm{MSA}}=9\cdot H\cdot W\cdot C_{1}+9\cdot H\cdot W\cdot \left(3\cdot C+48\right)$$

## 4 EXPERIMENTAL RESULTS

### 4.1 Dataset

Two different datasets were used in this study. The first dataset mentioned in this paper is used in the comparative experiment between MSA‐DDCovidNet network and some state‐of‐the‐art CNNs. CXR images in the above dataset come from two datasets: Kaggle CXR dataset [20] (https://www.kaggle.com/paultimothymooney/chest‐xray‐pneumonia) and the dataset collected by Joseph et al. [21]. Kaggle CXR dataset has a total of 5863 images, including pneumonia and normal CXR images. From the above two classes of images, 4265 images and 1575 images were selected. The dataset proposed by Joseph et al. has a total of 790 CXR images and CT images of patients infected with COVID‐19 or other pneumonia. Finally, 412 CXR images of with COVID‐19 patients are selected in this dataset. Therefore, the experimental dataset in this article contains a total of 6252 images. 310 COVID‐19 images, 1341 normal images, and 3875 pneumonia images are randomly selected from the experimental dataset as the training set. The remaining 102 COVID‐19 images, 234 normal images, and 390 pneumonia images are used as the test set.

In the following section, COVIDx dataset [22] is adopted to verify the performance of MSA‐DDCovidNet on other CXR image datasets. The COVIDx dataset is obtained according to the dataset generation method provided by Wang et al. [22], and finally got 589 COVID‐19 images, 8851 normal images and 6053 images of pneumonia. Similar to the method of Nihad et al. [23], 100 COVID‐19 images, 885 normal images, and 594 pneumonia images in COVIDx are randomly selected as the test set, and the remaining as the training set.

**FIGURE 4:**
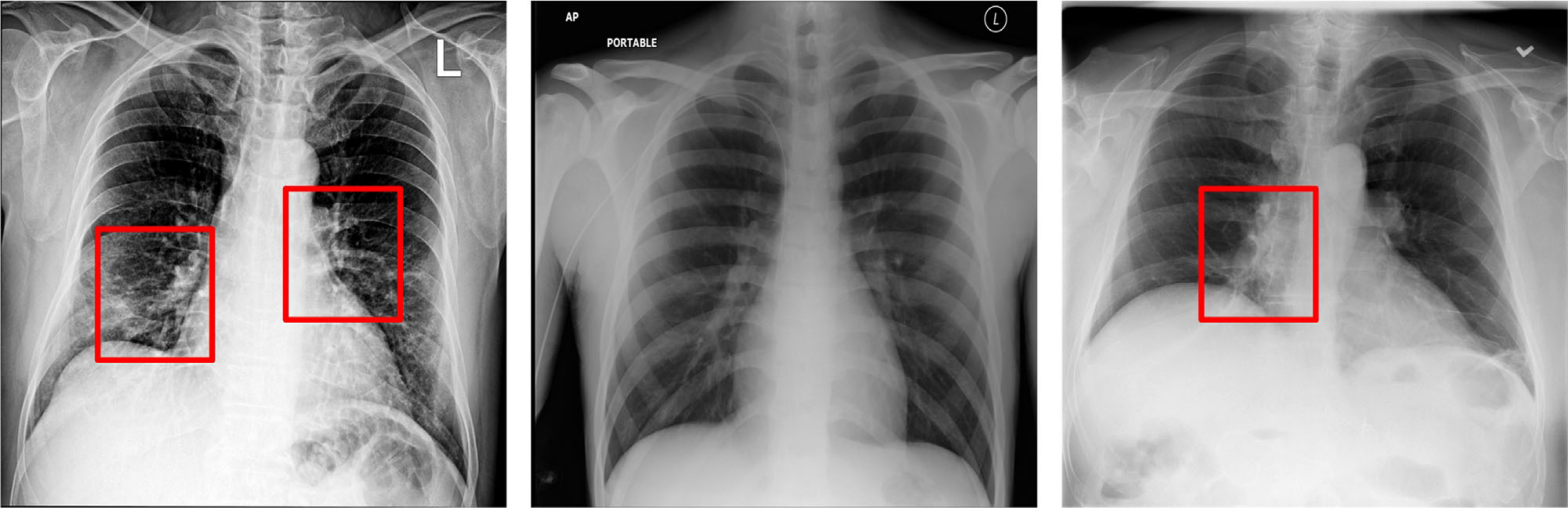
Cases of CXR Images. (a) Represent COVID‐19 CXR images. CXR images of COVID‐19 are mainly characterized by Pulmonary interstitial edema and exudation, thickening of pulmonary grain and multiple patchy and spotted shadow (b) Represent normal CXR images. (c) Represent pneumonia CXR images

Figure 4 shows an example of various CXR images in the experimental dataset of this work. It can reflect the high inter‐class similarity and low intra‐class variance of CXR images, which ratchet up the difficulty to the CXR images classification task.

### 4.2 The evaluation criteria of model

In terms of model evaluation criteria, we refer to the evaluation criteria adopted by most medical image classification models. Accuracy, precision, sensitivity, specificity, F1‐score, receiver operating characteristic (ROC) curve and area under the curve (AUC) are adopted as the model evaluation criteria. Some of the formulas for these evaluation criteria are as follows:

$$\mathrm{Accuracy}=\frac{T_{P}+T_{N}}{T_{P}+T_{N}+F_{p}+F_{N}}$$

$$\mathrm{Precision}=\frac{T_{p}}{T_{p}+F_{p}}$$

$$\mathrm{Sensitivity}=\frac{T_{p}}{T_{p}+F_{N}}$$

$$\mathrm{Specificity}=\frac{T_{N}}{T_{N}+F_{p}}$$

$$F1-\mathrm{score}=\frac{2\;\mathrm{TP}}{2\;\mathrm{TP}+\mathrm{FP}+\mathrm{FN}}$$

In these equations, T_P_ denotes true positive, F_P_ means false positive, F_N_ represents false negative, and T_N_ represents true negative.

### 4.3 Preprocessing and parameter settings

Since model training requires sufficient data samples, data augmentation techniques are used in this work. First, the resolution of the CXR images is scaled to a fixed size of 256 × 256, and the centre crop is applied to make the size 224 × 224. Then we perform a series of data enhancement processing on the training set: flip the CXR images horizontally with a probability of 0.5, and then randomly adjust the brightness, contrast, and saturation of the images to 0.6–1.4 times. After data enhancement technology, in fact, the number of samples used for training is four times that of the training set. This article conducts all experiments in the same configuration environment. The software platform and hardware environment are shown in Table 1.

**TABLE 1:** Experimental platform configuration

Attribute	Configuration information
Operating system	Ubuntu 18.04.1
CPU	Intel(R) Xeon(R) CPU E5‐2670 v3 @ 2.30GHz
GPU	GeForce RTX 2080
CUDNN	CUDNN 7.5.0
CUDA	CUDA 10.0.130
Frame	Pytorch
IDE	Pycharm
Language	Python

After many experiments, the training strategy of this experiment is summarized. The initial learning rate of the experimental models was set to 0.001. Each group of experiments was trained 150 cycles of epoch, and the loss function was the Cross‐Entropy loss function for label smoothing regularization [24] with epsilon = 0.1. And Adam [25] optimizer with betas = (0.9, 0.999) is used to make the model converge quickly. The batch‐size of training set and test set are 32 and 16 respectively.

### 4.4 Experimental results and analysis

In order to illustrate the lightweight and classification performance of our proposed model, several state‐of‐the‐art models are used as the control group in the experiments, such as VGG19 [26], GoogLeNet [27], ResNet50 [28], DenseNet121 [6]. The control group also contain various lightweight networks such as SqueezeNet1.0 [29], ShuffleNet [30], MobileNetV2 [18] and ShuffleNetV2 [31]. The performance of the above models is shown in Table 2. As can be seen from the Table 2, the classification accuracy, precision, sensitivity, specificity and F1 score of MSA‐DDCovidNet are 97.96%, 98.09%, 98.07%, 98.33% and 98.07%, respectively. Obviously, each criteria value of our proposed network is better than other networks. Taking the traditional network ResNet50 [28] in the control group as an example, its accuracy is 93.53%, which is the traditional network with the highest accuracy in our experiment. However, it is still 4.43% lower than the proposed network.

**TABLE 2:** Values of criteria of experimented models

Model	Accuracy (%)	Precision (%)	Sensitivity (%)	Specificity (%)	F1‐score (%)
VGG19 [26]	93.11	96.09	92.93	96.47	93.02
GoogleNet [27]	92.56	95.29	91.56	95.78	92.06
ResNet50 [28]	93.53	96.01	93.15	96.53	93.34
DenseNet121 [6]	93.11	95.98	92.75	96.38	92.92
SqueezeNet1.0 [29]	67.91	45.83	50.51	64.16	57.93
MobileNet [4]	88.53	90.14	87.25	91.84	87.89
ShuffleNet [30]	87.02	90.08	86.17	92.31	86.59
MobileNetV2 [18]	89.26	91.89	88.51	93.16	88.89
ShuffleNetV2 [31]	92.01	91.92	91.74	96.29	91.87
MSA‐DDCovidNet	**97.96**	**98.09**	**98.07**	**98.33**	**98.07**

In terms of the network complexity, it can be seen from the Table 3 that the parameter and the amount of calculation of MSA‐DDCovidNet outperform the other methods. Taking the lightweight networks ShuffleNet [30] and SqueezeNet1.0 [29] as examples, they are the networks with the least amount of calculation and parameters in the control group respectively. But they are still not as lightweight as our network, and their classification performance is also far less than our network. Moreover, as shown in Table 3 the parameters and the amount of calculation of ResNet50 [28] are 54.68 and 43.21 times that of ours respectively, which is obviously not as light‐weight as MSA‐DDCovidNet.

**TABLE 3:** Parameters and flops of several deep learning models and MSA‐DDCovidNet

Model	Flops (million)	Params (million)
VGG19	18 736.81	137.04
GoogLeNet	1 434.21	5.32
ResNet50	3 919.13	22.42
DenseNet121	2 731.91	6.62
SqueezeNet_1.0	702.71	0.73
MobileNet	560.73	3.11
ShuffleNet	142.02	0.91
MobileNetV2	311.13	2.13
ShuffleNetV2	144.72	1.22
**MSA‐DDCovidNet**	**90.69**	**0.41**

**FIGURE 5:**
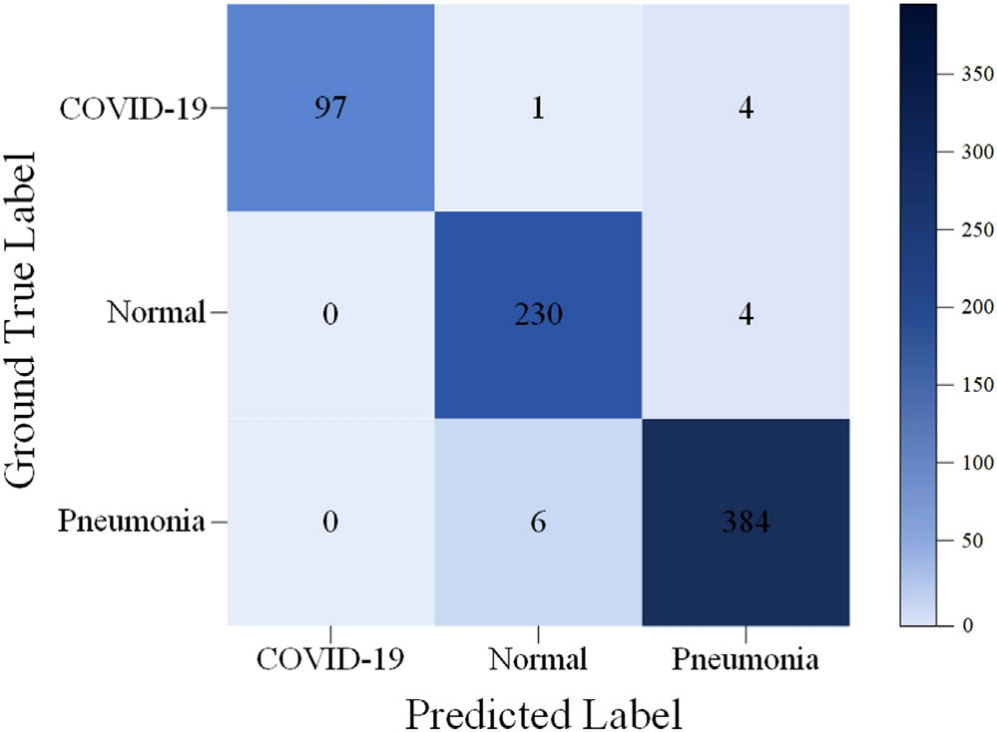
The confusion matrix of MSA‐DDCovidNet

Figure 5 shows the confusion matrix of MSA‐DDCovidNet on test set. As can be seem from Figure 5, the sensitivity of COVID‐19 is 95.10% when 97 images are detected from 102 tested images. In addition, the true detection of the Normal class is 98.29%. Further, the Pneumonia class achieves 98.46% success ratio. Based on this confusion matrix, the values of various criteria of MSA‐DDCovidNet are calculate, as shown in Table 4. As shown in Table 4, the weighted average precision, sensitivity, and specificity of MSA‐DDCovidNet are all higher than 97%, which are 97.95%, 97.93% and 98.23% respectively. More notably, the precision and specificity of MSA‐DDCovidNet to recognize COVID‐19 reach 100%. Since the baseline sensitivity of Covid‐19 CXR images is 69% [32], it proves that our proposed network can effectively improve the diagnostic efficiency of COVID‐19.

**TABLE 4:** Precision, sensitivity, specificity of MSA‐DDCovidNet on test set

Class	Precision (%)	Sensitivity (%)	Specificity (%)
COVID‐19	100	95.10	100
Normal	97.05	98.29	98.58
Pneumonia	97.96	98.46	97.55
Average	97.95	97.93	98.23

In addition, some deep learning methods for detection of COVID CXR images are compared with MSA‐DDCovidNet, as shown in Table 5. As is shown in Table 5, DarkCovidNet [14] has the fewest parameters among the five comparison models. But it is still 2.68 times more than that of MSA‐DDCovidNet, and its classification accuracy is 10.94% lower than MSA‐DDCovidNet. ECOVNet‐Soft [23] has the highest accuracy among the five comparison models, which is still 2.26% lower than our proposed network, and its parameter is 12.146 times that of our proposed network. Therefore, considering the network performance and complexity, it demonstrates that our proposed network is a recommendable intelligent method for recognizing CXR images of COVID‐19.

**TABLE 5:** Comparison of MSA‐DDCovidNet with other deep learning methods developed using X‐ray images

Method	Numbers of cases	Model	Accuracy/%	Params (Million)
Rahimzadeh et al. [12]	224 COVID‐19 700 Pneumonia 504 Normal	XResNet50V2 [12]	92.85	45.37
Wang et al. [22]	358 COVID‐19 5 538 Pneumonia 8066 Normal	Covid‐Net [22]	93.3	11.75
Khan et al. [9]	284 COVID‐19 657 Pneumonia 310 Normal	CoroNet [9]	94.59	33.00
Ozturk et al. [14]	125 COVID‐19 500 Pneumonia 500 Normal	DarkCovidNet [14]	87.02	1.10
Nihad et al. [23]	589 COVID‐19	ECOVNet‐Soft [23]	95.70	4.98
	8851 Pneumonia			
	6053 Normal			
Our Method	412 COVID‐19 4 265 Pneumonia 1575 Normal	MSA‐DDCovidNet	97.96	0.41

The results of these excellent methods are obtained in different datasets. If these methods are verified with the same data set, and the performance differences will be more intuitive and convincing. In order to further verify the effectiveness of MSA‐DDCovidNet, an experiment is supplemented with COVIDx [22] dataset: The performance of the six models in Table 5 in COVIDx [22] dataset under the experimental environment and parameter settings of this study (see Section 4.3 for details) will be observed and compared. The results of the above experiments are shown in Table 6.

**TABLE 6:** Values of criteria of experimented models

Model	Accuracy (%)	Precision (%)	Sensitivity (%)	Specificity (%)	F1‐score (%)
XResNet50V2	80.87	75.69	80.87	81.87	78.19
Covid‐Net	93.22	93.19	93.22	93.79	93.17
CoroNet	**94.81**	**94.85**	**94.81**	**95.45**	**94.78**
DarkCovidNet	74.86	70.14	74.86	76.24	72.42
ECOVNet‐Soft	86.83	81.26	86.83	87.20	83.92
MSA‐DDCovidNet	90.63	90.86	90.63	92.51	90.65

As shown in Table 6, CoroNet, proposed by Khan et al. [9], outperforms the other models in all criteria. Based on Xception [10], CoroNet [9] adopts deep separable convolution to reduce the parameters of the model, instead of standard convolution. However, the large depth and width of the network result in a mass of parameters. Covid‐Net [22] makes full use of point convolution and depth separable convolution in the PEPX module, which effectively reduces the parameters, and finally obtains a better performance with fewer parameters. XResNet50V2 [12] by Rahimzadeh et al. contains two parallel sub‐networks: Xception [10] and ResNet50V2 [11], and adopts a fully connected layer to classify the features extracted by these two sub‐networks, which produces a mass of parameter. Moreover, its complex structure makes it difficult to optimize. Therefore, in the end, it needs more parameters, but it can't get good performance. The structure of DarkCovidNet [14] is similar to VGGNet [26], consisting of some standard convolutional layers, max pooling layers and fully connected layers. It has fewer parameters with low depth and width, which makes it difficult to learn a relatively large data set, like COVIDx. Therefore, DarkCovidNet [14] performs poorly in this experiment. After the experimental preprocessing, the CXR images in COVIDx are finally resize to 224 × 224. For better comparison, the ECOVNet‐Soft in this experiment is based on the EfficientNet‐b0 model, rather than the original EfficientNet‐b5. The ECOVNet‐Soft obtained by this method is a relatively lightweight network, and its performance in this experiment is slightly different from that in the original paper [23]. Such difference is considered reasonable due to the difference of hardware devices. MSA‐DDCovidNet is the model with the fewest parameters in the experiment. Due to the application of deep separable convolution, feature reuse and multi‐scale feature fusion, it still performs well in this experiment. From a comprehensive point of view, although CoroNet [9] and Covid‐Net [22] have achieved better performance with sophisticated designs, their parameters are more than 28 times that of MSA‐DDCovidNet. Moreover, MSA‐DDCovidNet can perform better than those more complex models such as XResNet50V2 [12], DarkCovidNet [14], ECOVNet‐Soft [23].

**FIGURE 6:**
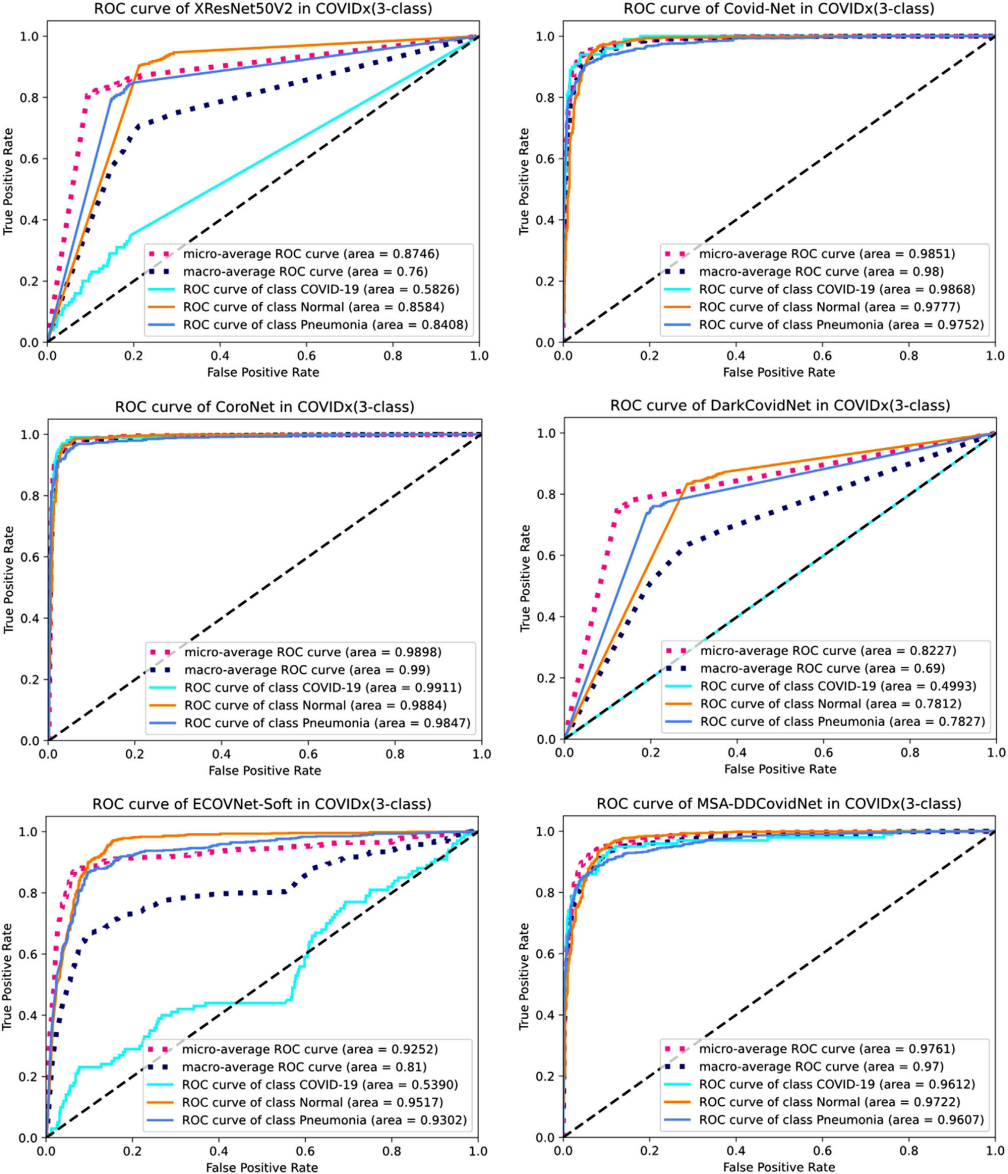
ROC curves of MSA‐DDCovidNet and the other deep learning models in Table 5

ROC curve is considered as an effective evaluation method that reflects the classification performance of the model. It can reflect the trade‐off between the true positive rate and the false positive rate. Figure 6 shows the ROC curves of the six models. The labels in Figure 6 show the micro and macro average and class‐wise AUC scores.

The comparison results of Figure 6 are similar to those in Table 6. Both CoroNet [9] and Covid‐Net [22] have better ROC curves and AUC values, and the performance of MSA‐DDCovidNet is only behind these two networks. It can also be found in Figure 6 that the three underperforming networks – XResNet50V2, DarkCovidNet, and ECOVNet‐Soft – have poor classification capabilities for COVID‐19. In the same experimental settings with the other models, DarkCovidNet underperforms. The intuitive explanation is that its low depth and width make it difficult to detect relatively few Covid‐19 CXR images among the numerous CXR images. In contrast, MSA‐DDCovidNet has achieved a relatively well performance with fewer parameters. In summary, MSA‐DDCovidNet is a network worthy of being applied to CXR image recognition.

## 5 DISCUSSION

**FIGURE 7:**
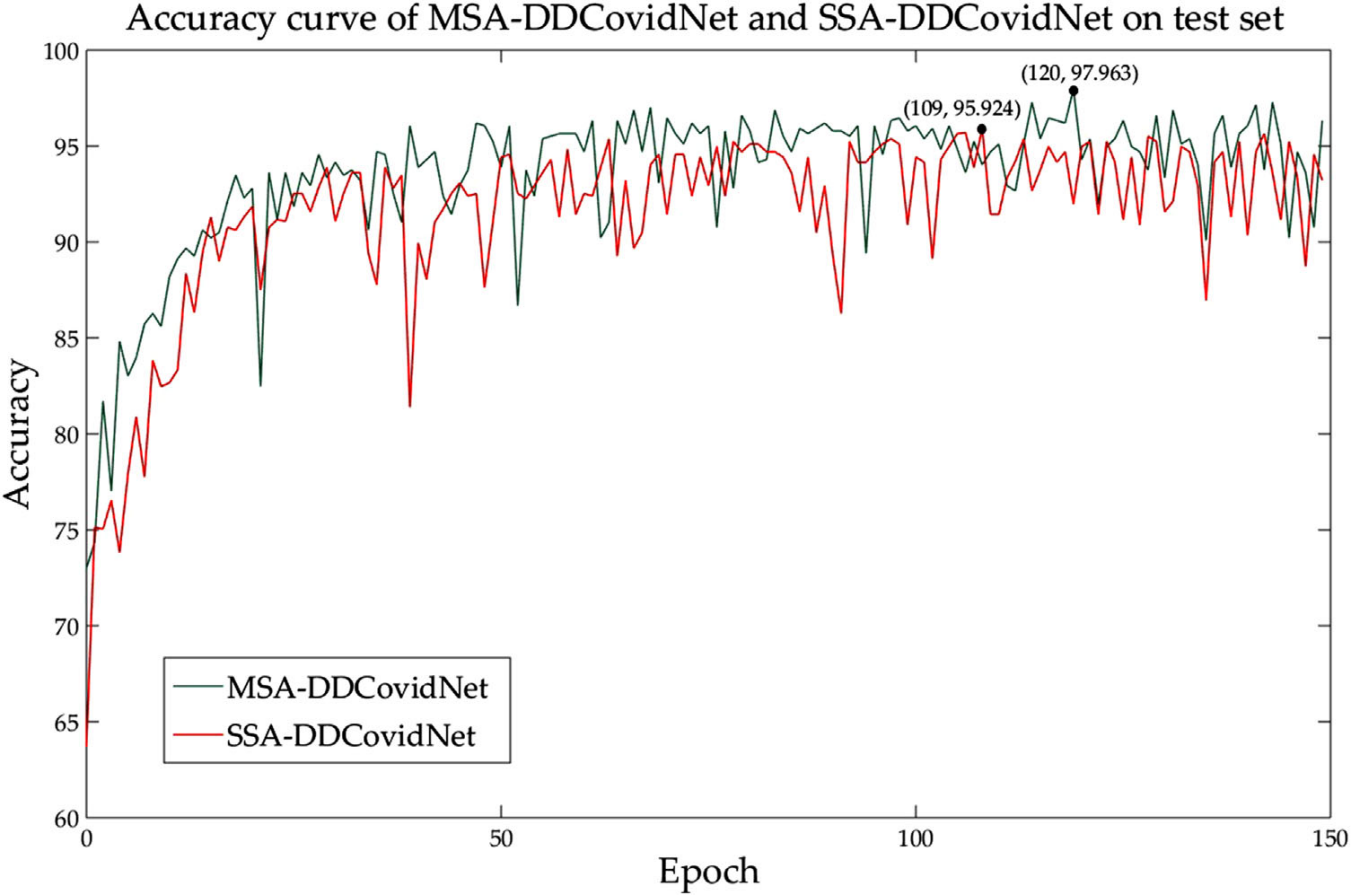
Accuracy curves of MSA‐DDCovidNet and SSA‐DDCovidNet on test set. The red line represents the accuracy curve of MSA‐DDCovidNet and the green line represents the accuracy curve of SSA‐DDCovidNet. The two curves peaked at the 120th epoch and the 109th epoch, respectively

In order to verify that the multi‐scale spatial attention mechanism is better than the traditional spatial attention mechanism, a network SSA‐DDCovidNet is designed as the control group. In the SSA‐DDCovidNet, the attention mechanism in MSA‐DDCovidNet is replaced with the traditional single‐scale spatial attention mechanism to obtain SSA‐DDCovidNet. Figure 7 shows the accuracy curves of the two networks in the experimental dataset (). As can be seen from Figure 7, the average accuracy of the proposed network in 150 epochs is higher than that of SSA‐DDCovidNet, and the highest accuracy is 2.03% higher than that of SSA‐DDCovidNet.

**FIGURE 8:**
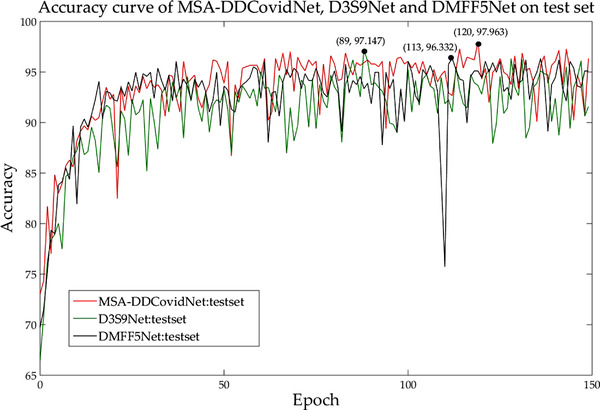
Accuracy curves of MSA‐DDCovidNet, D3S9Net and DMFF5Net on test set. The red line represents the accuracy curve of MSA‐DDCovidNet, the green line represents the accuracy curve of D3S9Net and the black line denotes the accuracy curve of DMFF5Net. The three curves peaked at the 120th epoch, the 89th epoch and the 113th epoch respectively

An additional experiment is conducted to verify the need for obtaining spatial attention map. Two networks are designed in this experiment: D3S9Net and DMFF5Net as comparison networks. In MSA‐DDCovidNet, the output feature map of the 1st DMFF module is used to generate spatial attention map. While in D3S9Net, the output feature map of the 9th D3S module is used to generate spatial attention map. Similarly, in DMFF5Net, the output feature map of the 5th DMFF module is used to generate the attention map. 1st DMFF module, 5th DMFF Module and 9th D3S Module are in the shallow, middle and deep layers of the network respectively. Different depth feature maps are adopted to generate attention maps and then compare their performance. The test accuracy curves of the three networks are shown in Figure 8. Our interpretation of this result is that in each down sampling, the feature map will lose some spatial information. Since the features in the shallow feature map are not compressed many times, the included features are relatively complete. Therefore, it is more reasonable to obtain the spatial attention map in the shallow layer of the network.

As a lightweight network, MSA‐DDCovidNet gets great advantages from its structure. But its performance still has a gap with some sophisticated and highly complex networks. The model needs further study and improvement in the future. And MSA‐DDCovidNet will be rescaled in the further work, under the premise of ensuring the lightweight of the network, using more parameters for better performance.

## 6 CONCLUSION

In this paper, to recognize COVID‐19 CXR images effectively, two kinds of feature sensitive modules proposed by our team are used: DMFF module and D3S module. Based on these two modules and MSA mechanism, we proposed MSA‐DDCovidNet with strong spatial representation capacity and few parameters. To verify the performance of our proposed network, two datasets are adopted. In the preliminary experiment, 4265 CXR images of pneumonia patients, 1575 normal CXR images and 412 CXR images of COVID‐19 patients are selected from two datasets. The performance of our network is compared with a series of other networks through experiments. The results of the preliminary experiment show that MSA‐DDCovidNet has excellent performance, and its classification accuracy for test set is 97.96%. More notably, its precision, sensitivity and specificity for COVID‐19 are 100%, 95.10% and 100%, respectively. In addition, a larger dataset COVIDx is also adopted to verify the performance of MSA‐DDCovidNet. An additional experiment is designed and the performance of MSA‐DDCovidNet is compared with some other deep learning models. Finally, MSA‐DDCovidNet got a good performance. Two additional ablation experiments are also conducted to verify the effectiveness of MSA mechanism. Therefore, it's believed that using MSA‐DDCovidNet to detect COVID‐19 CXR can effectively improve the diagnostic efficiency, and help detect and isolate patients in time. Due to the shortage of COVID‐19, it's necessary to collect more COVID‐19 CXR images to better illustrate the effectiveness of our proposed network. Although MSA‐DDCovidNet performed very well in the experiment, it still needs further clinical research and testing. After further training and testing, MSA‐DDCovidNet is expected to be put into practical application in auxiliary diagnosis COVID‐19.

## CONFLICTS OF INTEREST

The authors declare that they have no conflicts of interest.

## Data Availability

All data sets are public data sets that can be downloaded online.
